# The Role of Point-of-Care Ultrasound in the Emergency Department in the Diagnosis and Management of Infective Endocarditis

**DOI:** 10.7759/cureus.63107

**Published:** 2024-06-25

**Authors:** Munzir Malik, Abdul Aziz, Muhammad S Farooqi, Mujtaba Mohammed, Irfan A Rind

**Affiliations:** 1 Acute Medicine, Wrexham Maelor Hospital, Wrexham, GBR; 2 Internal Medicine, Insight Hospital and Medical Center, Chicago, USA; 3 Cardiology, Wrexham Maelor Hospital, Wrexham, GBR

**Keywords:** pocus (point-of-care ultrasound), bicuspid aortic valve disease, aortic valve insufficiency, splenic infarct, cardio thoracic surgery, infective endocarditis

## Abstract

Infective endocarditis (IE) is a serious cardiovascular condition with the potential to lead to severe valvular regurgitation. We present a case of a 65-year-old male who presented with a fever and was diagnosed with IE through point-of-care ultrasound (POCUS). The patient's condition subsequently led to severe aortic regurgitation. Timely diagnosis facilitated by POCUS played a crucial role in the management of this case. The patient underwent successful timely surgical intervention to prevent further infective embolism and heart failure due to severe acute aortic regurgitation. This case underscores the pivotal role of POCUS in the early diagnosis and multidisciplinary management of cardiology diseases, highlighting its importance in delivering optimal patient care.

## Introduction

Infective endocarditis (IE) is a serious cardiovascular condition that warrants an early diagnosis. If left untreated, it can lead to severe cardiovascular complications like valvular regurgitation, and prosthetic valve dysfunction like dehiscence, paravalvular abscess, and fistula. Complications are not limited to the heart, it can lead to thromboembolic incidents leading to stroke, cerebral abscess, splenic infarcts, etc. [1].

We present a case of a 51-year-old male who presented with a splenic infarct and a new cardiac murmur. A point-of-care ultrasound (POCUS) was performed by the on-call acute medical team which led to the diagnosis of IE. Timely diagnosis facilitated by POCUS played a crucial role in the management of this case. The patient underwent a timely successful aortic valve replacement and experienced a remarkable recovery. This case underscores the pivotal role of POCUS in the early diagnosis and multidisciplinary management of cardiology diseases, highlighting its importance in delivering optimal patient care.

## Case presentation

A 51-year-old male with a past medical history of hypertension and type 2 diabetes mellitus presented to the emergency department with abdominal pain and fever. He subsequently had a CT abdomen in the emergency department which revealed a splenic infarct. Physical examination revealed a diastolic murmur over the left sternal edge in the third intercostal space but no signs of heart failure or any peripheral signs of IE [2].

Given the clinical suspicion of endocarditis due to splenic infarction, fever, and a murmur, a POCUS was performed at the bedside, which revealed a large echogenicity attached to the aortic valve associated with significant aortic regurgitation (Videos 1, 2).

**Video 1:** Parasternal long-axis view showing a large (2.5 x 0.5 cm) vegetation on the aortic valve

**Video 2:** Apical five-chamber view with color Doppler showing severe aortic regurgitation

Diagnosis

The POCUS findings were consistent with IE therefore given a large vegetation and thromboembolic mechanism like splenic infarction, an urgent cardiology referral was made from the emergency department. Furthermore, blood cultures were obtained, and echocardiography was performed to assess the severity of valvular involvement. A transesophageal echocardiogram (TOE) confirmed aortic valve vegetation measured 2.4 cm x 0.5 cm. It also revealed a bicuspid aortic valve which predisposes to IE (Videos 3, 4).

**Video 3:** TOE at mid-esophageal level showing bicuspid aortic valve TOE: transesophageal echocardiogram

**Video 4:** TOE confirming the diagnosis of large aortic valve vegetation and ruling out any extension to the aortic root (aortic root abscess) TOE: transesophageal echocardiogram

Laboratory findings

This gentleman on initial presentation had markedly raised CRP of 108 (normal value <5). His white blood cells were within the normal range with mildly raised neutrophils of 8.7 x10^9/L (normal range 1.7-7.5). His liver function and kidney function tests were unremarkable. Blood cultures grew positive for bacilli (*Abiotrophia defectiva*). *A. defectiva* is a rare cause of IE which is mostly found in the oral cavity and the intestines and can be a cause of culture-negative endocarditis as it is very difficult to isolate and needs special media for culture [3]. We were advised by microbiology to treat him with Ceftriaxone and Gentamycin which resulted in significant improvement in his CRP. His chest X-ray and ECG were unremarkable.

Management

The patient was promptly started on broad-spectrum antibiotics and managed in consultation with the cardiothoracic surgery team. Due to the severity of aortic regurgitation and the risk of complications, including heart failure and embolic events, the decision was made to proceed with surgical intervention.

Surgical intervention

**Figure 1:**
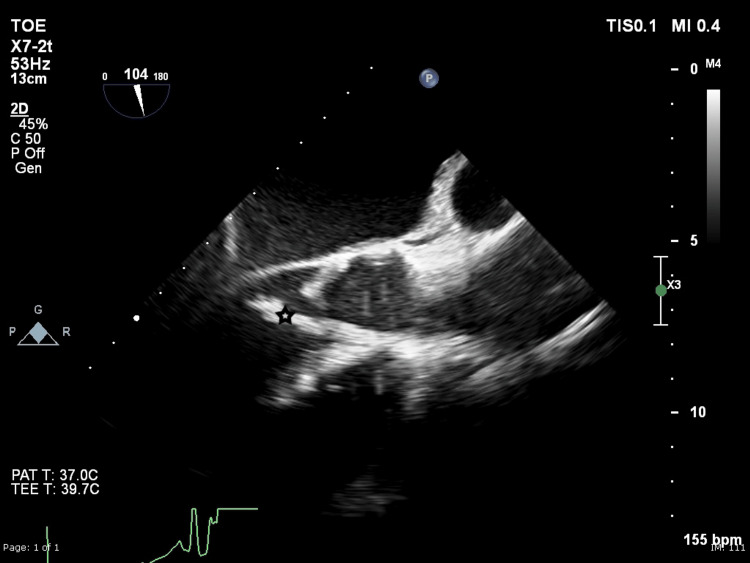
Intraoperative TOE shows a large aortic valve vegetation denoted by a star TOE: transesophageal echocardiogram

**Figure 2:**
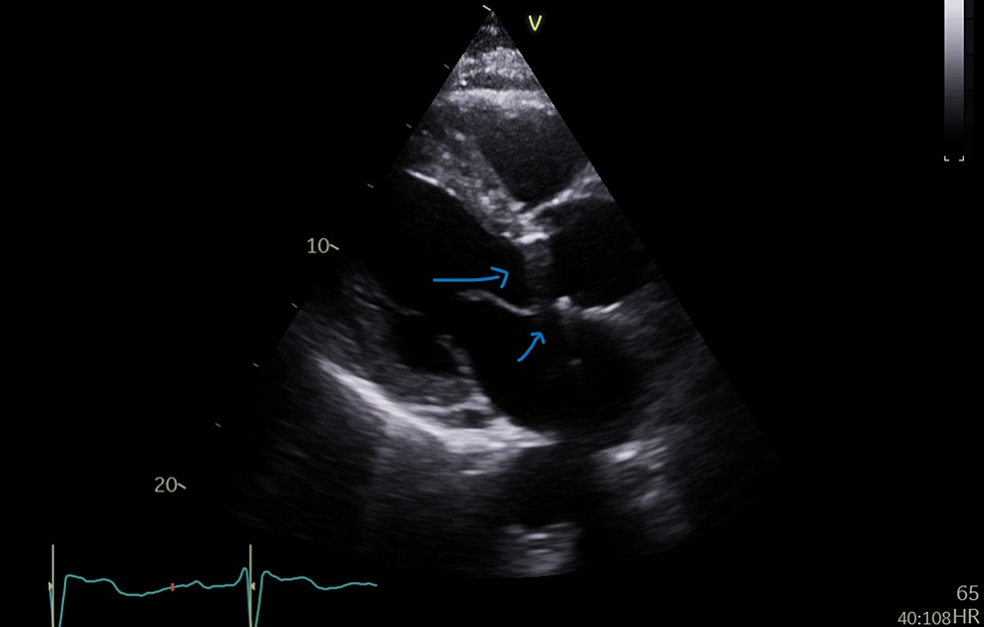
Reverberation artifact coming from the metallic aortic valve (blue arrows)

The patient underwent metallic aortic valve replacement after the excision of the vegetation. Figure 1 shows intraoperative findings of a large aortic valve vegetation. Figure 2 shows post-operative findings of the prosthetic aortic valve after the complete excision of vegetation. Postoperative recovery was uneventful and the patient remained asymptomatic. He was discharged on Ceftriaxone for six weeks post valve replacement as per the initial blood culture sensitivities which grew *A. defectiva*.

Follow-up

At the follow-up visit, he had an uneventful recovery with no residual valve lesion on echocardiography. The patient was also advised on antibiotic prophylaxis for dental procedures as per current guidelines.

## Discussion

In this case, the diagnosis of IE leading to severe aortic regurgitation was made by the utilization of POCUS. POCUS is a bedside imaging technique that provides real-time information about cardiac structures [3]. In this case, the presence of vegetation on the aortic valve was visualized using POCUS, which led to the early diagnosis of IE. Transthoracic echocardiography has a central role in the identification of vegetation and associated complications. The presence of vegetation is a major criterion to diagnose IE as per Duke's criteria. This timely identification of the culprit pathology allowed for the initiation of appropriate prophylactic antibiotic therapy, addressing the infection at its source. It also helped the medical team to urgently organize cardiology reviews and subsequent specialized investigations like a TOE [4]. POCUS not only confirmed the diagnosis of IE but also assessed the severity of mitral regurgitation. The ability to assess the degree of valvular involvement is crucial in determining the urgency of surgical intervention. Other indications for surgery include severe heart failure, invasion beyond the valve leaflets, recurrent systemic embolization, large mobile vegetations, or persistent sepsis despite adequate antibiotic therapy for more than 5-7 days [5]. In this case, severe aortic regurgitation was noted, necessitating surgical correction to prevent complications such as heart failure and embolic events given a large vegetation [6].

IE is a complex cardiovascular condition that often requires a multidisciplinary approach for optimal management. In this case, the collaboration between cardiology and cardiothoracic surgery teams was vital. The decision to perform surgery was made based on the assessment of the TTE and TOE findings of severe aortic regurgitation with large vegetation, demonstrating the importance of integrating imaging data into treatment decisions [7].

The patient's successful surgical intervention highlights the effectiveness of early diagnosis and timely action [8]. Post valve replacement, there was no residual aortic regurgitation on the transthoracic echocardiogram.

The management of IE extends beyond the surgical intervention [9]. The patient's postoperative care included a course of antibiotics, and they were advised to continue antibiotic prophylaxis for dental and invasive procedures as recommended by guidelines. This emphasizes the need for ongoing surveillance and adherence to long-term management strategies to prevent recurrent infection [10,11].

## Conclusions

In conclusion, this case report underscores the significance of POCUS in the diagnosis and management of cardiology diseases, particularly IE. POCUS not only facilitated the early diagnosis of IE but also provided critical information about the severity of valvular damage, guiding the decision for surgical intervention. The successful outcome in this case demonstrates the importance of a coordinated and multidisciplinary approach in treating complex cardiovascular conditions, ensuring the best possible patient care and recovery.
